# Ulcerating Ileocolitis in Severe Amatoxin Poisoning

**DOI:** 10.1155/2015/632085

**Published:** 2015-08-18

**Authors:** Matthias Peter Hilty, Marcel Halama, Anne-Katrin Zimmermann, Marco Maggiorini, Andreas Geier

**Affiliations:** ^1^Medical Intensive Care Unit, University Hospital of Zurich, Rämistrasse 100, 8091 Zurich, Switzerland; ^2^Department of Gastroenterology and Hepatology, University Hospital of Zurich, Rämistrasse 100, 8091 Zurich, Switzerland; ^3^Department of Pathology, University Hospital of Zurich, Rämistrasse 100, 8091 Zurich, Switzerland

## Abstract

Amatoxin poisoning is still associated with a great potential for complications and a high mortality. While the occurrence of acute gastroenteritis within the first 24 hours after amatoxin ingestion is well described, only very few descriptions of late gastrointestinal complications of amatoxin poisoning exist worldwide. We present the case of a 57-year-old female patient with severe amatoxin poisoning causing fulminant but reversible hepatic failure that on day 8 after mushroom ingestion developed severe abdominal pain and watery diarrhea. Ulcerating ileocolitis was identified by computed tomography identifying a thickening of the bowel wall of the entire ileum and biopsies taken from the ileum and large bowel revealing distinct ileitis and proximally accentuated colitis. The absence of discernible alternative etiologies such as infectious agents makes a causal relationship between the ulcerating ileocolitis and the amatoxin poisoning likely. Diarrhea and varying abdominal pain persisted over several weeks and clinical follow-up after six months showed a completely symptom-free patient. The case presented highlights the importance to consider the possibility of rare complications of *Amanita* intoxication in order to be able to respond to them early and adequately.

## 1. Introduction

*Amanita* is a genus of fungi in the phylum of basidiomycota consisting of around 600 known species [1]. Many of them are toxic and are easily misrecognized macroscopically by untrained individuals such as amateur mushroom hunters, leading to 205 cases of reported poisonings in Europe between 1971 and 1980, with an overall mortality of 22.4%, in the subpopulation of children less than 10 years even of 51.3% [2]. Incidence may have increased to date to around 50 per year in Europe [3] and somewhat less in the USA [4]. In Europe, mainly* Amanita phalloides* poisoning is described. Toxicity is attributable to amatoxins, a group of several polypeptides composed of around 35 amino acids that act as inhibitors of eukaryotic RNA polymerase II [2, 5] and therefore inhibit mRNA synthesis, leading to cell death. Through first-pass effect and a high level of metabolism, hepatocytes as a cell population are mainly affected, but there are also reports of kidney toxicity [6] and rarely late gastrointestinal toxicity [7]. Clinically, amatoxin poisoning initially manifests with gastroenteritis and an apparent recovery after 24 to 36 hours, coinciding with the onset of dose-dependent fulminant hepatic and multisystem organ failure [8].

## 2. Case Report

**Figure 1:**
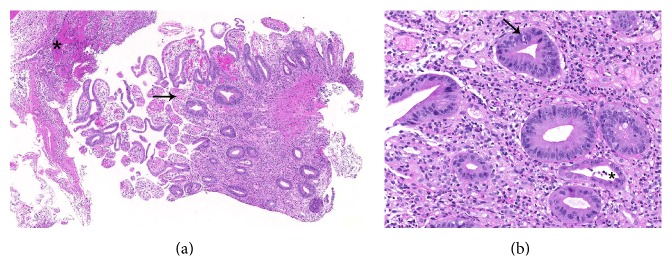
Biopsy of the ileum. (a) Biopsy of the ileum with acute ulcerating inflammation and fibrinoleukocytic exudate (*∗*). Enteric architecture is changed with only fragmented residual villi and loss of Paneth cells. Mucosal vessels are heavily dilated (→) (40x magnification). (b) The lamina propria is filled by a dense mixed inflammatory infiltrate. Some neutrophilic granulocytes invade the epithelium of the crypts (→). In the lower right corner of the picture a residual crypt with atrophic epithelium and intraluminal cell detritus can be seen (*∗*). The epithelial cells show regeneratory and reactive changes with enlargement of the nuclei and prominent nucleoli (200x magnification).

**Figure 2:**
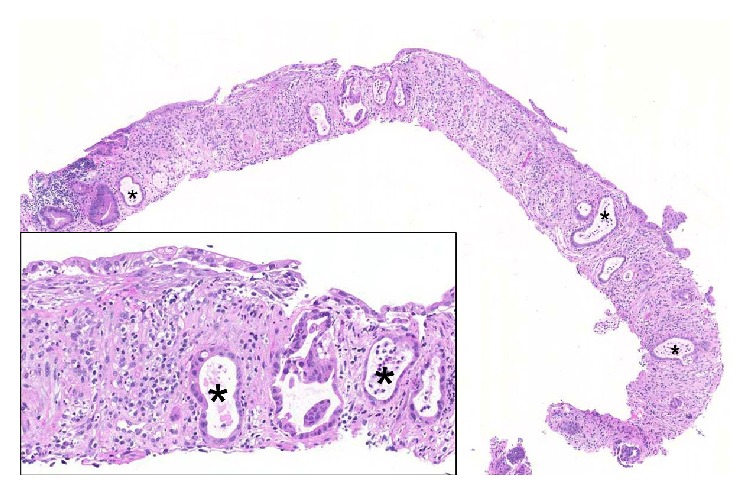
Biopsy of the cecum, showing rarefication and atrophy of the crypts, dilation of the lumina with intraluminal cell detritus (*∗*), and fibrosis of the lamina propria. Epithelial cells present reactive, respectively, regeneratory nuclear atypia in the context of inflammation (40x magnification); for details see the inset (200x magnification).

A 57-year-old female patient presented on day 3 after ingestion of approximately 200 g of self-collected mushrooms with a history of severe gastroenteritis beginning on day 1 and lasting through admission. On the day of admission, she became increasingly weak, providing her with the reason to seek medical attention. Otherwise, the patient had no medical record of any significance. The patient presented with stable cardiovascular and pulmonary function, afebrile, with diffuse abdominal tenderness and lively bowel sounds. There were no mushroom residues available for examination by an expert, but* Amanita phalloides* intoxication, which was highly compatible with the description of the patient and the clinical course, was proven via detection of *α*-amanitin in the urine using ELISA (Buehlmann Laboratories, Schonenbuch, Switzerland) with a maximal concentration of 42.2 *μ*g/L on admission on day 3. Directly on admission, intravenous silibinin (Legalon SIL, Rottapharm, Monza, Italy) and acetylcysteine (Fluimucil 10%, Zambon Pharma, Milan, Italy) and multiple dose oral activated charcoal therapy were initiated at the intensive care unit according to current guidelines [9]. Silibinin was administered intravenously at 20 mg/kg per day for three days after a loading dose of 5 mg/kg; acetylcysteine was administered intravenously at 150 mg/kg over 1 hour, followed by 12.5 mg/kg over 4 hours and 6.25 mg/kg over 16 hours. At admission on day 3, liver enzymes were already elevated, reaching maximum on day 4 with alanine aminotransferase levels of 6894 U/L, aspartate aminotransferase of 5232 U/L, and lactate dehydrogenase of 10596 U/L. Alkaline phosphatase and *γ*-glutamyl transferase levels remained normal. As a measure of liver function, factor V activity was nondetectable on day 4, with factor II activity at 29% (60–150) and factor VII activity at 25% (60–150); the international normalized ratio (INR) was 2.7. Fortunately, liver function improved without further interventions on day 7 (Table 1). The patient initially also presented with renal failure. Renal ultrasonography studies were normal; however a calculated fractional excretion of urea of 65% hinted at an intrarenal pathology. Renal failure was completely reversible by day 4 without need for renal replacement therapy. On day 8, however, the patient developed severe abdominal pain, tenderness on examination with signs of peritonitis especially in the lower right quadrant, accompanied by watery diarrhea and an elevation of inflammatory parameters. Cardiovascular and pulmonary function remained stable. Abdominal computed tomography showed a thickening of the bowel wall of the entire ileum and no other pathologies. On day 12 after mushroom ingestion gastrointestinal biopsies were taken from the ileum and the large bowel; see Figures 1 and 2. Diagnostic findings were most prominent in the mucosa of the ileum and the cecum and decreased towards the rectum. The mucosa of the ileum showed, in addition to acute cryptitis and ulcerating inflammation with fibrinoleukocytic exudate (pseudomembranes), regenerating changes of the epithelium. Acute erosive and ulcerating inflammatory changes together with a rarefication and atrophy of crypts, fibrosis of the lamina propria, and regenerating epithelial changes were most prominent in the cecum. These findings of a distinct ileitis and proximally accentuated colitis are not specific. Architectural and regenerating epithelial changes and fibrosis indicate a sustained or previous damage of the mucosa. Screening for Clostridium difficile toxin using ELISA as well as cultures for Clostridium difficile and* Salmonella*,* Shigella*, and* Campylobacter* species remained negative. With supportive therapy, gastrointestinal symptoms as well as inflammatory markers showed a tendency to regression so that the patient could be dismissed in good shape on day 17 after ingestion of* Amanita*. Diarrhea and varying abdominal pain persisted over several weeks thereafter. Clinical follow-up after six months showed a completely symptom-free patient.

## 3. Discussion and Conclusions

Worldwide, only a few cases of late gastrointestinal complications of* Amanita* intoxication have been reported [7]. Supported by the absence of other possible etiologies such as the detection of infectious agents, in our reported case, the impressive manifestation of ulcerating ileocolitis may be directly due to amatoxins, which could be detected in high concentrations in the patient's urine. Even though urine toxin concentration does not correlate as well with the clinical manifestations of the intoxication as the amount of toxin ingested per body weight [2], patient history also indicates a fairly high amount of ingested fungus, as 50 g of* Amanita phalloides* may already contain a lethal dose of toxin for an adult. Still, other possible causes of ulcerating ileocolitis cannot entirely be excluded even in the absence of known triggers such as nonsteroidal anti-inflammatory drugs or known ischemia. Renal failure can be the result of direct kidney damage due to amatoxins [6] as is at least in part suggested by the increased fractional excretion of urea; however, a combined prerenal etiology is likely in light of the rapid and complete amelioration following adequate volume management. Specific treatment to* Amanita* intoxication is available and was administered early in our case. Silibinin, an extract of the Mediterranean milk thistle (*Silybum marianum*), acts as specific inhibitor of amatoxin uptake into hepatocytes and may improve survival [10]. N-Acetyl cysteine, presumably acting through its antioxidant effects, has also recently been shown to significantly improve survival [11]. Finally, multiple dose activated charcoal administered until at least day 4 after ingestion removes the amatoxins from the enterohepatobiliary recirculation and thereby improves total toxin clearance [12]. Plasma exchange has also been discussed [13, 14], but there is little data about the risk-benefit-ratio. The effect of all of these treatment modalities on late gastrointestinal complications however is, to our knowledge, unknown. Clinical knowledge about the possibility of late gastrointestinal manifestations of* Amanita* intoxication was probably more common in the seventh and eighth decade of the twentieth century [15, 16], even though it remains important to generate and distribute knowledge about it, especially since* Amanita* intoxication is a permanent threat to the population and still remains difficult to treat. Also, further research is needed to possibly draw a connection between amatoxins and direct action on the bowel and to understand the specific mechanisms that may be involved. The case presented highlights the importance to consider the possibility of rare complications of* Amanita* intoxication in order to be able to respond to them early and adequately.

## Figures and Tables

**Table 1:** Toxin quantification and inflammatory and organ function parameters at admission with treatment initiation with silibinin and fluimucil and during the initial three days (I + 3 through I + 5), the onset of abdominal symptoms (I + 8), and dismissal from hospital (I + 17). Note the peak of liver injury and dysfunction at I + 4 with rapid recovery and the marked increase in inflammatory parameters at onset of abdominal symptoms.

Days after ingestion	I + 3 hospital admission, treatment initiation	I + 4	I + 5	I + 8 abdominal symptoms	I + 17 dismissal from hospital
Alpha-amanitin (urine) [*μ*g/L]	42.4	2.9	Below threshold (<1.5)	—	—
C-reactive protein [mg/L]	15	24	36	85	5
Lactate [mmol/L]	2.3	2.4	1.1	—	—
Creatinine [*μ*mol/L]	274	94	64	67	83
Blood urea nitrogen [mmol/L]	17.6	14.8	9.6	3.2	1.7
Aspartate-aminotransferase [U/L]	1249	5235	2998	727	30
Alanine-aminotransferase [U/L]	1380	6894	5430	120	19
Factor V [%]	35	Below threshold (<10)	49	121	138
INR [1]	1.6	2.7	1.7	1.2	1.1
